# Review of genotoxicity biomonitoring studies of glyphosate-based formulations

**DOI:** 10.3109/10408444.2015.1010194

**Published:** 2015-02-17

**Authors:** Larry D. Kier

**Affiliations:** ^a^ Private Consultant, Buena Vista, CO, USA

**Keywords:** biomonitoring, formulation, genotoxicity, glyphosate, mutagenicity

## Abstract

Human and environmental genotoxicity biomonitoring studies involving exposure to glyphosate-based formulations (GBFs) were reviewed to complement an earlier review of experimental genotoxicity studies of glyphosate and GBFs. The environmental and most of the human biomonitoring studies were not informative because there was either a very low frequency of GBF exposure or exposure to a large number of pesticides without analysis of specific pesticide effects. One pesticide sprayer biomonitoring study indicated there was not a statistically significant relationship between frequency of GBF exposure reported for the last spraying season and oxidative DNA damage. There were three studies of human populations in regions of GBF aerial spraying. One study found increases for the cytokinesis-block micronucleus endpoint but these increases did not show statistically significant associations with self-reported spray exposure and were not consistent with application rates. A second study found increases for the blood cell comet endpoint at high exposures causing toxicity. However, a follow-up to this study 2 years after spraying did not indicate chromosomal effects. The results of the biomonitoring studies do not contradict an earlier conclusion derived from experimental genotoxicity studies that typical GBFs do not appear to present significant genotoxic risk under normal conditions of human or environmental exposures.

**Abbreviations**
BC: blood cells
BM: blood monocyte cells
BNMN: binucleated cells with micronuclei
CBMN: cytokinesis-block micronucleus
CA: chromosomal aberrations
GBF: glyphosate-based formulation
MN: micronucleus
MOMN: mononuclear cells with micronuclei
SCE: sister chromatid exchange
8-OHdG: 8-hydroxydeoxyguanosine

## Introduction

Glyphosate is the active ingredient of very extensively used herbicide formulations and, accordingly, glyphosate and glyphosate-based formulations (GBFs) have been extensively studied for their toxic properties. One of these toxic properties is genotoxicity and there has been a recent extensive review of glyphosate and GBF experimental genotoxicity studies (Kier and Kirkland 2013). This review concluded that there was a strong weight of evidence that glyphosate and GBFs are predominantly negative in well-conducted core bacterial reversion and *in vivo* mammalian micronucleus and chromosomal aberration assays. Although some positive results for glyphosate and GBFs were reported in DNA damage assays and for the micronucleus endpoint for GBFs in non-mammalian studies, the positive results were associated with high dose levels and/or toxic effects. The preponderance of negative results in core assays supports the conclusion that reports of DNA damage or non-mammalian micronucleus effects are likely to be secondary to cytotoxicity rather than indicative of DNA-reactive mechanisms. This conclusion is consistent with and supported by a recent review of 14 experimental rodent carcinogenicity studies of glyphosate that indicated a weight of evidence that there was no carcinogenic effect related to glyphosate treatment (Greim et al. 2015).

The earlier Kier and Kirkland (2013) review focused on experimental studies and did not consider reports of human or environmental biomonitoring studies where there was GBF exposure. This review complements the earlier review by identifying and considering a number of human and environmental biomonitoring studies where exposure to GBFs was indicated and one or more genotoxicity endpoints were employed. Such studies can provide perspective on potential for effects on humans or other organisms with actual environmental or occupational exposures. However, they are also much more complicated to interpret and derive definitive conclusions from than experimental studies because of confounding exposures to other agents, complexity of applying methodology to subject populations and limits on availability of endpoints and sample sizes.

## Identification of published studies

The published studies for review consideration were identified by literature searches for published reports containing references to glyphosate or GBFs (e.g., Roundup^™^ formulation) that also contained searchable terms which indicated that human or environmental genotoxicity studies were performed (e.g., alkaline single cell gel electrophoresis (comet) or micronucleus endpoints). Emphasis was placed on publications in peer-reviewed journals. Abstracts or other sources with incomplete information were not considered. Reviews without original data were not considered for evaluation; however, these reviews were examined to determine if there were any cited publications that had not been detected in the literature searches.

## General methodology

### Populations

Table 1 summarizes the identified genotoxicity biomonitoring studies involving GBF exposure. Most of these studies are cross-sectional studies in which genotoxicity biomarkers in an exposed population were compared to an unexposed referent population. A few studies are longitudinal studies where sampling was made before and after exposures (Lebailly et al. 2003, Bolognesi et al. 2009). For cross-sectional studies, a suitable sample size and a carefully matched referent population are important (Albertini et al. 2000, Collins et al. 2014). Although sample size should ideally be defined in reference to a pre-determined desired sensitivity, this does not appear to have been rigorously considered in the identified studies. A few of the studies had quite small (e.g., < 25) exposed and referent population sizes (e.g., Gregio D’Arce and Colus 2000, Vlastos et al. 2006, Paz-y-Mino et al. 2007, Bortoli et al. 2009).

**Table 1.** Studies of human and environmental populations with reported GBF exposure.

Exposed population^a^	Endpoint^b^	Pesticide/GBF exposures	Exposed group result^c^	References
*Human studies*				
Agricultural workers (20); R (16)	Lymphocyte CA^NC^	19 pesticides reported used Including GBF	No statistically significant increase in CA	Gregio D’Arce and Colus (2000)
Greenhouse farmers (104); R (44)	Lymphocyte SCE^NC^	9 pesticides or pesticide classes reported as used. GBF used by 99/104 farmers	Statistically significant increases in SCE/chromosome and high SCE frequency cells	Shaham et al. (2001)
Floriculturists (107); R (61)	Lymphocyte CBMN	> 30 pesticides reported used. GBF use reported in 57/107 workers	Statistically significant increase in BNMN	Bolognesi et al. (2002)
Hungarian agricultural workers (84); R (65)	Lymphocyte CBMN Buccal MN	14 pesticides reported used. GBF use frequency reported as 16.1%	No statistically significant increases in BNMN or buccal cell MN frequencies	Pastor et al. (2003)
Fruit growers (12 in one season for urine and comet; 17 in second season for urine only) ; NR	BM comet^NC^ Ames test on urine	Samples collected before and after captan spraying. GBF use reported in 2/29 growers 1 day before captan spraying and in 1/19 grower on the day of captan spraying	No statistically significant effects on comet % DNA damage or tail moment; correlation between predicted captan exposure and response In Salmonella strain TA102	Lebailly et al. (2003)
Floriculturists (51); R (24)	Lymphocyte CBMN	25 pesticides reported used. GBF use reported in 21/51 workers with average of 106.5 kg/year applied	No statistically significant increase in BNMN	Bolognesi et al. (2004)
Workers exposed to pesticides (33); R (33)	Lymphocyte SCE Lymphocyte CBMN Lymphocyte CA	> 30 pesticides reported used including GBF	Statistically significant increases in BNMN and SCE but not CA	Costa et al. (2006)
Farmers (11); R (11)	Lymphocyte CBMN^NC^	17 pesticides reported used. GBF use reported in 3/11 farmers	Statistically significant increase in MN frequency but not in frequency of BNMN; statistically significant increases in small MN	Vlastos et al. (2006)
Fruit farmers (29); NR	BC DNA adducts (^32^P-postlabelling)	GBF use reported in 1 of 29 fruit farmers. Sampling on morning of and morning after spraying	No statistically significant effects comparing relative adduct levels at different sampling times	Andre et al. (2007)
Individuals at or near GBF aerial spraying (24); R (21)	BC comet^NC^	GBF aerially sprayed within 3 km. Blood samples collected two weeks to two months after spraying	Statistically significant increase in comet tail length and appearance of high damage comets	Paz-y-Mino et al. (2007)
Workers exposed to pesticides (54); R (30)	BC comet	13 pesticides reported used including GBF	Statistically significant increase in damaged cells	Simoniello et al. (2008)
Humans in 3 areas where GBF was sprayed (60, 64 and 28); R (region of no pesticide exposure, 60).	Lymphocyte CBMN	Samples collected before, within 5 days and 4 months after GBF spraying in 3 regions Pesticide use reported by 76.6%, 61.7%and 28.6% of subjects in GBF sprayed regions	Statistically significant increase in BNMN sampled within 5 days of GBF spraying in 3 regions; statistically significant decrease In 4 month sample compared to < 5 day sample in 1 region.	Bolognesi et al. (2009)
Agricultural workers (29); R (37)	Buccal MN	10 pesticides reported used including GBF	Statistically significant increase in MN cell frequency	Bortoli et al. (2009)
Agricultural workers (70); R (70)	Lymphocyte SCE Buccal MN	25 pesticides reported used including GBF	Statistically significant increases in SCE/metaphase and MN cell frequency	Martinez-Valenzuela et al. (2009)
Subjects in areas with GBF aerial spraying up to 2 years previously (92); R (90)	Lymphocyte CA^NC^	Aerial GBF spraying for illicit crop control up to two years before sampling	Normal karyotypes and percentage of chromosomal fragility within normal parameters	Paz-y-Mino et al. (2011)
Agricultural workers (81); R (46)	BC comet Buccal MN^NC^	25 pesticides reported used including GBF	Statistically significant increases in damaged comets and MN cell frequency	Benedetti et al. (2013)
Children living in areas of pesticide application (125); R (125)	Buccal MN^NC^	> 30 pesticides reported used including GBF	Statistically significant increase in MN cell frequency	Gomez-Arroyo et al. (2013)
Agricultural workers (41); R (32)	BC comet^NC^ Buccal MN^NC^	Exposure of up to 7 different pesticides with 56.7% of workers exposed to a single pesticide (fenpropathrin, carbofuran or GBF)	Statistically significant increase in MN cell frequency and in comet endpoints (%DNA in tail and tail moment)	Khayat et al. (2013)
Pesticide sprayers (80); R (206)	BC 8-OHdG	> 30 pesticides used including GBF	Statistically significant increases in 8-OHdG; no statistically significant increase with frequency of GBF applications in last spraying season	Koureas et al. (2014)
*Environmental Studies*				
Meadow voles living on golf courses (22 in 2001, comet only; 61 in 2002, comet and MN); R (0 in 2001; 8 in 2002)	BC comet^NC^ Erythrocyte MN^NC^	Numerous pesticides reported used including GBF	Comet tail length and moment statistically correlated with total pesticide exposure in 2001 but not 2002; no statistically significant pesticide effects on polychromatic erythrocyte MN frequencies	Knopper et al. (2005)
Fish from dams (various species; 3 per species)	Erythrocyte MN	Wide GBF use reported in adjacent lands along with other pesticides	Higher MN frequencies than normal or expected from other reports but no negative concurrent controls used	Salvagni et al. (2011)

^a^Description of exposed population with number of exposed individuals in (). R with () indicates number of individuals in non-exposed referent population. NR indicates no concurrent referent population studied.

^b^Genotoxicity endpoint(s) measured. See abbreviations for endpoint abbreviations. ^NC^ after SCE, CBMN or comet endpoints indicates that slides were not indicated as coded before scoring.

^c^Results reported for exposed group compared to referent group.

Careful matching of exposed and referent populations for cross-sectional studies requires consideration of the specific endpoint and confounding factors that might affect the endpoint. Recommendations of major endpoint specific factors include gender and age for the CBMN endpoint (Battershill et al. 2008, Fenech et al. 2011), age for the buccal micronucleus (MN) endpoint (Bonassi et al. 2011), and gender, age and smoking status for the comet endpoint in blood cells (Collins et al. 2014). For genotoxicity endpoints, a large number of other factors may also be considered as possible confounding variables such as diet (Bonassi et al. 2011, Fenech et al. 2011, Collins et al. 2014), sleep (Kahan et al. 2010, Tenorio et al. 2013), disease status (Albertini et al. 2000, Battershill et al. 2008, Fenech et al. 2011), and seasonal variation (Albertini et al. 2000, Moller 2005, Verschaeve et al. 2007).

Many of the human biomonitoring studies had similar gender, age and usually smoking and alcohol consumption distributions for their exposed and referent populations. Although many of the studies indicated that information on lifestyle or other factors was collected (e.g., medical history and treatments, X-ray exposures and diet), most of the studies did not present comprehensive detailed data on these confounding factors. Some of the studies had moderate to fairly large differences in gender distribution (Bolognesi et al. 2002, 2004, Pastor et al. 2003, Simoniello et al. 2008, Benedetti et al. 2013, Koureas et al. 2014). One factor recommended for recording of the blood cell comet endpoint in human biomonitoring studies is exercise (Collins et al. 2014); however, the cross-sectional studies employing the comet endpoint did not appear to explicitly consider this as a confounding variable.

### Exposures

Human exposures were usually characterized by self-reporting of the types of pesticides used as determined by survey of the exposed population or by more general use information. Additionally, the use of personal protective equipment may have been indicated. In most cases pesticides were characterized only by the active ingredient and not as a specific formulation. In some cases the extent of individual pesticide use was described as a frequency of use and/or amount of use but in most cases there were exposures to multiple pesticides. There are only a few biomonitoring studies where some assessment of the specific effects of exposures to GBFs can be inferred from the circumstances or exposure data presented. The identified studies only rarely attempted to estimate actual amount of exposure to specific pesticides or to evaluate exposure by chemical monitoring. No cases of chemical monitoring of exposure to glyphosate or GBFs were encountered in the genotoxicity biomonitoring studies. Uncertainty in extent and amount of exposure and dose is a major limitation in interpretation of the genotoxicity biomonitoring studies of pesticide exposure.

### Endpoints

The most common endpoints employed in the biomonitoring studies were the CBMN assay on cultured lymphocytes (six human studies), the micronucleus assay on buccal cells (six human studies) and the comet assay on blood cells (five human studies and one environmental study). Other endpoints included measurement of sister chromatid exchange (SCE) in cultured lymphocytes (three human studies), chromosomal aberration in cultured lymphocytes (three human studies), erythrocyte micronucleus assays (two environmental studies), and bacterial reversion (Ames test strains) on urine (one human study). Two human studies measured DNA alterations (bulky adducts and oxidative DNA damage).

The CBMN assays generally used similar standardized methodologies for culture, including addition of cytochalasin B at 44 h after phytohemagglutinin stimulation. The studies used whole blood rather than isolated leukocytes for culture and scored 1000 or 2000 binucleated cells per subject for micronuclei. Referent population frequencies of binucleated cells with micronuclei (BNMN) ranged from about 1.8 to 9 per 1000 which seems reasonably close to a mean of 6.5 per thousand with an inter-quartile range of 3–12 per thousand observed for a large number of normal subjects from many laboratories (Fenech et al. 2011).

The buccal micronucleus (buccal MN) assays generally followed recommendations for number of cells scored with 1000–3000 cells scored per subject. There is a recommendation for the use of DNA-specific staining for this assay such as Feulgen-Fast Green (Thomas et al. 2009). Two of the laboratories used relatively non-specific Giemsa staining (Benedetti et al. 2013, Bortoli et al. 2009). The mean frequencies of micronucleated cells in referent populations ranged from about 0.37 per thousand to 1.78 per thousand. This range seems reasonably close to a mean of 0.74 micronucleated cells per thousand for a large number of healthy subjects not knowingly exposed to genotoxic substances or radiation (Bonassi et al. 2011). The study with the highest mean frequency of micronucleated cells in a referent population (1.78 per thousand) employed the relatively non-specific Giemsa stain (Bortoli et al. 2009).

The comet studies generally used similar standard methodology for cell lysis, alkaline treatment, and staining of DNA. One study used isolated leukocytes (Lebailly et al. 2003) but the other studies used whole blood. It should be noted that whole blood contains a high percentage of short-lived neutrophils and thus may be more suitable for recent exposures to genotoxic agents (Collins et al. 2014). Recent guidance for comet assay methodology suggests that the most useful comet measurement is the percentage of DNA in the comet tail (Anderson et al. 2013, Azqueta and Collins 2013, Collins et al. 2014). Only one of the six comet studies reported measurement of percentage of DNA in the comet tail (Khayat et al. 2013).

Most of the endpoints employed in the biomonitoring studies involve visual scoring for endpoints or visual selection of comets for image analysis. There are consistent and numerous recommendations that slides for scoring for these endpoints should be coded so that the scorer is not aware of the treatment conditions, individual or groups to which the slides belong (e.g., OECD 479, 1986, OECD 474, 1997, Albertini et al. 2000, Tice et al. 2000, Hartmann et al. 2003, Fenech 2007, Thomas et al. 2009, OECD 475, 2014, OECD 489, 2014). However, a number of the biomonitoring studies for these endpoints, as indicated in Table 1, did not include an explicit statement in the methodology that slides were coded for analysis. It is possible that the methodology used actually did involve coding of slides but that this was not mentioned in the publication. If this is the case then clear indication of coding slides for analysis should be encouraged in the methodology sections of such publications. Alternately, it is possible that coding was not used and that the scorers may have been aware of the groups to which the slides belonged. This would be a significant deviation from recommended practice and coding of slides and reporting this in the methodology should be encouraged for all biomonitoring study endpoints where visual scoring or selection of objects is involved.

## Results for human biomonitoring studies

### Studies with low GBF exposure incidence

Table 2 summarizes conclusions about the studies relevant to GBF effects. For some of the human biomonitoring studies, the indicated frequency or incidence of pesticide exposure to GBF in the pesticide exposed population was very low (Pastor et al. 2003, Lebailly et al. 2003, Vlastos et al. 2006, Andre et al. 2007). The incidence of GBF exposure reported for these studies was too low to allow any reasonable conclusions about any relationships between GBF exposure and genotoxicity endpoint effects or lack of effects.

### Studies with exposure to multiple pesticides

A number of human monitoring studies in Table 1 and as summarized in Table 2 indicated exposure to a list of multiple pesticides including GBF but did not indicate the frequency or extent of exposure to any specific pesticides (Gregio D’Arce and Colus 2000, Costa et al. 2006, Simoniello et al. 2008, Bortoli et al. 2009, Martinez-Valenzuela et al. 2009, Benedetti et al. 2013, Gomez-Arroyo et al. 2013). One of the studies did not find statistically significant increases for the lymphocyte CA endpoint in agricultural workers (Gregio D’Arce and Colus 2000). The other six studies reported statistically significant increases for genotoxic endpoints for pesticide exposed populations compared to referent populations. An interesting observation of the Costa et al. (2006) study is that two endpoints (lymphocyte CBMN and SCE) had statistically significant increases in the exposed population but the chromosomal aberration endpoint did not. This suggests the possibility of different sensitivity to genotoxic effects of the endpoints which could possibly reflect different mechanisms and sensitivities to those mechanisms. Some support for this possibility is also provided by the negative lymphocyte CA result of Gregio D’Arce and Colus (2000), but this study did not measure other endpoints. None of these studies presented any detailed information on individual pesticide exposure or ascribed observed genotoxic effects to any specific pesticide. The fact that there were exposures to multiple pesticides, ranging from 10 to more than 30, in these studies and an unknown extent or frequency of exposure to GBFs does not allow any conclusions about genotoxic biomarker effects or lack of effects related to GBF exposure. It should be noted that positive results in genotoxicity biomonitoring studies involving multiple pesticide exposures have been frequently observed regardless of whether these exposures included GBF (Bolognesi et al. 2003, Bull et al. 2006).

**Table 2.** Summary GBF exposure conclusions from human genotoxicity biomonitoring studies.

Study reference	GBF conclusions and comments^a^
*Reported low GBF exposure incidence*	
Pastor et al. (2003)	Not informative because of low reported incidence of GBF exposure
Lebailly et al. (2003)	Not informative because of low reported incidence of GBF exposure. Longitudinal study focusing on captan exposure
Vlastos et al. (2006)	Not informative because of low reported incidence of GBF exposure
Andre et al. (2007)	Not informative because of low reported incidence of GBF exposure. Longitudinal study with no referent population
*Multiple pesticide exposures and unknown extent of GBF exposure*	
Gregio D’Arce and Colus (2000)	Not informative because of exposures to multiple pesticides and unknown extent of GBF exposure. Negative result for CA endpoint indicates no positive effects from GBF exposure but extent of GBF exposure is not known
Costa et al. (2006)	Not informative because of exposures to multiple pesticides and unknown extent of GBF exposure. Negative results for CA endpoint indicates no positive effects from GBF exposure but extent of GBF exposure is not known
Simoniello et al. (2008)	Not informative because of exposures to multiple pesticides and unknown extent of GBF exposure
Bortoli et al. (2009)	Not informative because of exposures to multiple pesticides and unknown extent of GBF exposure
Martinez-Valenzuela et al. (2009)	Not informative because of exposures to multiple pesticides and unknown extent of GBF exposure
Benedetti et al. (2013)	Not informative because of exposures to multiple pesticides and unknown extent of GBF exposure
Gomez-Arroyo et al. (2013)	Not informative because of exposures to multiple pesticides and unknown extent of GBF exposure
*Multiple pesticide exposures and reported significant extent of GBF exposure*	
Shaham et al. (2001)	Not informative because significant exposures to multiple pesticides were reported including GBF. Positive SCE effects not ascribed to GBF exposure
Bolognesi et al. (2002)	Not informative because significant exposures to multiple pesticides were reported including GBF. Positive CBMN effects not ascribed to GBF exposure
Khayat et al. (2013)	Not informative because significant exposures to multiple pesticides were reported including GBF. Positive buccal MN and BC comet effects not ascribed to GBF exposure. Use of only one pesticide (including GBF) reported for a large proportion of the population but no separate endpoint analysis of single pesticide exposure indicated
*Informative for GBF exposure effects*	
Bolognesi et al. (2004)	Some limited evidence for lack of effects of GBF exposure on lymphocyte CBMN endpoint. No statistically significant increases in BNMN frequency of exposed population with significant proportion (21/51) reporting exposure to GBF. Difference in gender distribution between exposed and referent populations. Small sample size of population exposed to GBF
Paz-y-Mino et al. (2007)	Evidence for BC comet effects for population in region of GBF aerial spraying. Small exposed and referent populations with differences in gender distribution. Samples collected and processed at different times after spraying. No indication of coding of slides for scoring. Significant clinical signs of toxicity and much higher than normal rates of application reported for exposed population. Comet effects may be secondary to toxicity
Bolognesi et al. (2009)	Inconclusive for lymphocyte CBMN effects for populations in regions of aerial GBF spraying. Statistically significant increases in BNMN frequencies were observed immediately after GBF spraying but statistically significant correlations were not observed with self-reported exposure to spray and results were not consistent with GBF application rates
Paz-y-Mino et al. (2011)	Some evidence of lack of chromosomal effects in a population exposed earlier to GBF aerial spraying. Publication indicates no chromosomal effects but contains no details on methodology or detailed chromosomal aberration data
Koureas et al. (2014)	Some evidence of lack of oxidative DNA damage from GBF exposure. Univariate analysis indicated lack of statistically significant correlation between reported GBF exposure frequency and 8-OHdG in blood DNA. Exposures are reported from last spraying season and relationship between exposure and sampling is not clear

^a^See abbreviations for endpoint abbreviations.

Another set of human biomonitoring studies involved exposures to multiple pesticides but indicated frequency of exposure to specific pesticides that included a significant proportion of the exposed population using GBF (Shaham et al. 2001, Bolognesi et al. 2002, 2004, Khayat et al. 2013). One of these studies reported no statistically significant increase in BNMN frequency compared to a referent population for the CBMN endpoint in a population of 51 floriculturists of whom 21 reported GBF use (Bolognesi et al. 2004). Although the authors suggested trends for an increase in BNMN frequency with pesticide use and exposure time and a trend toward higher proportion of centromere-containing MN with pesticide exposure and in a subgroup using benzimidazolic compounds, the statistically negative result for BNMN frequency might be taken as some evidence indicating lack of detectable effect for this endpoint in the appreciable portion of floriculturists exposed to GBF.

Three other studies with multi-pesticide exposure including significant frequency of GBF use in the exposed populations reported positive genotoxic effects for the lymphocyte SCE endpoint (Shaham et al. 2001), the CBMN endpoint (Bolognesi et al. 2002), and the blood cell comet and buccal MN endpoints (Khayat et al. 2013). Two of these studies presented data on frequency of pesticide or pesticide class use and for both of these studies most participants used multiple pesticides and GBF use, while frequent, was not dominant compared to numerous other pesticides (Shaham et al. 2001, Bolognesi et al. 2002). Neither of these studies analyzed or attributed genotoxicity marker effects to specific pesticides and, given the multiplicity of pesticide exposures, there is no basis to conclude that GBF exposure was responsible for the effects observed. The Khayat et al. (2013) study reported that an appreciable percentage (56.7%) of the exposed population were exposed to only one pesticide and the single pesticide exposures were to GBF, fenpropathrin, or carbofuran. How many workers were exposed to each pesticide was not indicated. It should be noted that the Khayat et al. (2013) data table reporting multiplicity of pesticide exposures appeared to only present data for 30 workers but there were 41 workers in the exposed population. Despite the apparent occurrence of single pesticide exposures in a large portion of the exposed group, the study did not indicate a pesticide-specific analysis of genotoxic marker effects. In the absence of such analysis the genotoxic marker effects observed cannot be attributed to any specific pesticide, including GBF.

### Studies assessing GBF exposure effects

As indicated in Tables 1 and 2, there were four studies where specific information on GBF exposure effects was presented. Three published studies focused on populations believed to be exposed to GBFs by their presence at or near aerial GBF spraying operations (Paz-y-Mino et al. 2007, 2011, Bolognesi et al. 2009).

One of these studies reported induction of blood cell comet effects on a Northern Ecuadorian population living within 3 km of areas sprayed with GBF for illicit crop eradication (Paz-y-Mino et al. 2007). The sprayed material was reported to be Roundup Ultra, a GBF containing 43.9% glyphosate, polyethoxylated tallowamine surfactant, and a proprietary component, Cosmoflux 411F. The populations studied were relatively small (24 exposed individuals and 21 non-exposed individuals) and the referent population had a higher proportion of males (4/21 vs. 1/24 in the exposed group). Blood sampling was reported to have been at 2 weeks to 2 months after spray exposure and samples were indicated to have been processed immediately. Specific methods for collection, storage, and transport of blood samples were not described for either the exposed population or referent group but it was noted that referent group samples were not processed concomitantly with the exposed group samples. Time between collection and assay and storage conditions and variation in sampling time between exposed and referent sample collection have been cited as potentially important variables for human biomonitoring studies using the comet endpoint (Collins et al. 2014). Inclusion of reference standards is advised when samples are processed at different times (Azqueta and Collins 2013) but this was not indicated in Paz-y-Mino et al. (2007) publication. The Paz-y-Mino publication also did not indicate that slides were coded for scoring for comet effects. As noted above there are numerous recommendations for coding of slides scored in the comet assay unless the scoring is fully automated (Tice et al. 2000, Hartmann et al. 2003, Collins et al. 2014, OECD 489, 2014).

The Paz-y-Mino et al. (2007) study reported increases in damaged cell categories and statistically significant increases in DNA migration (tail length) in the presumably exposed population. Interpretation of the results of this study should consider numerous reported signs of toxicity in the exposed population and the reported application rate of 23.4 liters/ha which was stated to be more than 20 times the maximum recommended application rate. Some of the reported exposed group health effects described by Paz-y-Mino et al. (2007) appear to be consistent with severe exposures noted in clinical reports of acute poisoning incidents (often self-administered) with GBFs and other pesticide formulations rather than typical bystander exposures (Menkes et al. 1991). Given the considerably favorable general toxicology profile of glyphosate as reported by the WHO/FAO Joint Meeting on Pesticide Residues (WHO/FAO 2004) and in Williams et al. (2000), factors related to either high surfactant exposure, unusual GBF components in this formulation or other undocumented variables appear to be confounding factors in this study. It is possible that the reported comet effects, if indeed resultant from GBF exposure, could well have been secondary to the clinical toxicity reported in this study population.

Subsequent to the original Paz-y-Mino et al. (2007) study, a baseline study was conducted on residents on the northeastern Ecuadorian border near where there had been aerial applications of GBF (Paz-y-Mino et al. 2011). Apparently, samples were collected about 2 years after the last aerial spraying. The exposed population used for genomic and chromosome analysis (92 individuals) and the referent sample population (90 individuals) were much larger than those of the previous Paz-y-Mino et al. (2007) study and the proportion of males in the exposed population was much higher. Publication details on sample collection, storage, transportation, and methodology for chromosomal aberration analysis are very limited and typical data for the chromosomal aberration endpoint were not presented. Thus, there is some uncertainty that the endpoint used was the typical chromosomal aberration endpoint. Nevertheless, the publication indicated that none of the exposed population had any type of chromosomal alteration and the percentage of chromosomal fragility was within normal parameters.

Another publication (Bolognesi et al. 2009) reported results for a lymphocyte CBMN study of individuals in three areas of Columbia treated with GBF by aerial spraying for illicit crop eradication (Putumayo and Nariño regions) or sugar cane maturation (Valle del Cauca region). Other populations were from an area using manual eradication for illicit crops and pesticides including GBF for agriculture (Boyaca region) and a region where agricultural practices do not include pesticide application (Santa Marta region). Although the title of the publication contains the term “agricultural workers”, it appears that only some of the total population studied had agriculture as an occupation. The percent of subjects listing agriculture as an occupation varied from 7.1% in Valle del Cauca to 60% or more in Putumayo and Nariño. Although percentage of subjects reporting current use of pesticides is reported for the various regions and there was a reference to higher prevalence of use of genotoxic pesticides in Putumayo and Nariño no detailed information on the pesticides used or frequency of use was presented in the publication.

The human lymphocyte culture and scoring methodology employed in the Bolognesi et al. (2009) study appear to be generally consistent with commonly used and recommended practices for this assay. There is a question as to how long the blood samples used in the study were stored prior to initiating cultures. The publication only indicated that blood samples were kept at room temperature and cultures were initiated at a central laboratory within 24 h of collection. There may have been differences in the time between sampling and culture initiation for different sets of samples. Also, the populations in the aerially sprayed regions had a second sampling within 5 days after the first sampling and this second sampling time was not used for the other regions. It appears that collection and processing of samples may have occurred for different times for the aerially sprayed regions and the other regions.

The publication reported a small statistically significant increase in the frequency of BNMN in samples collected from people living in three regions within 5 days after spraying of GBFs compared with values for samples collected just before spraying. The publication also indicated a statistically significant increase of micronucleated mononuclear cells (MOMN) in the immediate post-spraying samples for two regions (Nariño and Valle del Cauca). In the samples taken 4 months after spraying, a statistically significant decrease in BNMN frequency compared to immediate post-spraying frequency was observed for one of the spraying regions (Nariño) but the other sprayed regions did not exhibit a statistically significant difference in BNMN frequency between the immediate post-spraying and 4-month samples.

Although the increases in BNMN frequencies in the post-spraying samples of the three regions suggest an effect from GBF exposure, more detailed consideration of exposure factors raises significant questions about this conclusion. The populations in each of the sprayed regions self-reported exposure to the spray (e.g., being in sprayed fields after spraying or observing spray drops in the air or on skin). For all three sprayed regions, there was no statistically significant difference in BNMN frequency between those self-reporting spraying exposure and those self-reporting no spraying exposure. The largest percentage post-spraying increase in BNMN frequency was reported for Valle del Cauca but only 1 of 26 people from this population self-reported spray exposure. Also, it was noted that GBF spraying in Valle del Cauca was at a rate significantly lower (1 kg acid equivalents glyphosate/ha) than that in Nariño and Putumayo (3.69 kg acid equivalents glyphosate/ha). The lack of clear correlation between self-reported exposure and BNMN increases after regional GBF spraying led to some caution in interpretation by the authors. The Bolognesi et al. (2009) publication suggested that results indicated low genotoxic risk from the GBF aerial spraying for illicit crop eradication. Another possible conclusion that appears to be supported by the self-reported exposure information is that this study does not clearly demonstrate an association between GBF exposure and CBMN endpoint effects.

Koureas et al. (2014) published a study examining effects of pesticide exposure on a measure of oxidative DNA damage, 8-hydroxydeoxyguanosine (8-OHdG) in blood DNA, which addressed whether GBF exposure appeared to affect this endpoint. The publication indicated that the exposed population had recently applied pesticides with no longer than 7 days between the last application and sampling. Several of the analyses were based on self-reported frequency of exposure to specific pesticides during the last spraying season and the timing relationship between specific pesticide applications and blood sampling is not clear. Statistically significant increases in 8-OHdG DNA levels were observed in blood samples collected from pesticide applicators compared to a non-exposed referent population. A univariate analysis was conducted to determine if specific high/low pesticide exposure classifications based on seasonal application frequencies were statistically associated with increased 8-OHdG levels in blood DNA. This analysis found statistically significant associations with 8-OHdG levels for herbicide exposure frequency and specifically for glufosinate herbicide exposure. Other statistically significant specific pesticide frequency exposure correlations were observed for neonicotinoids. A statistically significant exposure frequency correlation was not observed for GBF exposure. While certainly of limited power, this analysis provides some evidence that GBF exposures in pesticide applicators were not associated with oxidative DNA damage.

The human genotoxicity biomonitoring studies that specifically address GBF effects appear to have some evidence for lack of persistent genotoxic effects, especially under normal conditions of exposure. One study suggests lack of DNA oxidation effects with GBF application and a study employing CBMN does not show statistically significant effects correlating with self-reported exposure to GBF spraying. One study reported effects on the blood cell comet endpoint following exposures to very high levels of GBF spraying which apparently were sufficient to elicit significant clinical signs of toxicity. However, a subsequent study conducted 2 years after GBF spraying using much larger populations did not detect chromosomal alterations or an increase in chromosomal fragility indicating that the comet effects did not appear to be manifested as persistent genotoxic effects. It should be noted that there is growing appreciation that comet endpoint effects in biomonitoring studies may result from indirect (i.e., non DNA-reactive) mechanisms such as inhibition of DNA repair, perturbation of cytokinesis, and oxidative stress (Collins et al. 2014). It seems very likely that the observed blood cell comet effects, if indeed associated with GBF exposure, were secondary to toxicity from very high GBF exposures and that these effects do not indicate DNA-reactive genotoxicity or a genotoxic risk from normal GBF exposures.

## Results for environmental biomonitoring studies

There are two publications related to environmental biomonitoring for genotoxic endpoints. One study using blood cell comet and erythrocyte MN endpoints was conducted on samples from meadow voles living on or near golf courses where pesticides had been applied (Knopper et al. 2005). Different comet sample processing methodology (use or non-use of dimethylsulfoxide in lysis buffer) was used for the two different seasons and statistically significant differences in the average comet tail moment between the two seasons were ascribed to this different methodology. Although some suggestions of effects were reported, GBF was only one of a number of applied pesticides and the effects observed were considered by the authors as possibly attributable to exposure to Daconil^®^ fungicide.

A second publication reported results for the erythrocyte MN assay applied to fish collected from several dams in Brazil (Salvagni et al. 2011). GBF was one of a number of pesticides reported to be used in the area of the dams. This study reported what were considered to be high numbers of micronuclei in cells but there were no concurrent negative controls. In the absence of these controls, the results might not be interpreted as conclusively indicating effects of pesticide exposure.

## Conclusions

Two environmental genotoxicity biomonitoring studies conducted on a mammalian species and fish species were not informative about possible environmental genotoxic effects of GBFs. Both studies involved exposures or potential exposures to multiple pesticides without characterizing the relative extent of GBF exposure.

There have been a fairly large number of human genotoxicity biomonitoring studies where some exposure to GBFs was reported. Several of these studies were not informative about effects of GBF exposure because there was exposure to multiple pesticides and reported GBF exposure frequencies were low or very low. Another set of human biomonitoring studies were also not informative about possible genotoxic effects of GBF exposure because these studies listed exposure to large numbers of pesticides (10 to more than 30) in the exposed population without indicating the frequency or extent of exposure to any of the pesticides. Although positive genotoxic endpoint effects were observed in most of these studies no conclusions can be made regarding which pesticide exposures were responsible for the effects.

A third set of human genotoxicity biomonitoring studies involved exposures to multiple pesticides but did indicate significant frequency of GBF exposure in the populations. One of these studies did not find statistically significant effects for the lymphocyte CBMN endpoint in the exposed population compared to a referent population. This study offers some limited evidence for lack of significant, detectable effects on this endpoint for human exposure to any of the pesticides with significant exposure frequencies, including GBF, but the population sizes exposed were low. Three other studies reported positive genotoxic endpoint effects but the exposure data and endpoint data presented did not permit attribution of these effects to any specific pesticide exposure.

Finally, there are data from four human genotoxicity biomonitoring studies that provide information on GBF exposure effects. A study of oxidative effects on blood DNA indicated that observed increases in oxidative DNA damage did not statistically correlate with last season frequency of GBF application. These results provide limited evidence for this indirect genotoxic mechanism not operating at a significant level in humans using GBFs. Three studies involved measurement of genotoxic endpoints in human populations living in regions where GBFs were applied by aerial spraying. One study used a longitudinal design involving populations in regions of aerial GBF applications where samples were taken before, within 5 days and 4 months after GBF spraying. Statistically significant post-spraying increases for the CBMN endpoint were observed in these populations. However, the increases were not significantly correlated with self-reported exposure to the sprays or with the spraying application rate. Application of well-respected criteria for relating epidemiology cause and effect (Bradford-Hill 1965) to these results does not permit a conclusion that the observed effects were clearly related to GBF spray exposure. Two other studies were made of humans in GBF aerial spraying regions. A cross-sectional study found increases for the blood cell comet endpoint in the exposed population compared to a referent population. The exposures in this study appeared to be very excessive in terms of GBF application rate and significant signs of toxicity were observed in the exposed population. It seems possible that effects for this endpoint, if induced by GBF spraying exposure, may well have been indirect mechanism effects secondary to toxicity. A follow-up study of larger sample size from the sprayed regions conducted 2 years after spraying did not indicate any effects on chromosomal alteration or fragility endpoints. These latter results suggest that no persistent genotoxic effects were induced in the sprayed population and are consistent with the possibility that earlier reported comet effects may well have been secondary to toxic effects rather than resulting from a DNA-reactive mechanism.

The overall conclusion from the human biomonitoring studies is that none of the reported positive results for studies involving exposure to multiple pesticides present evidence specifically relating GBF exposure to these results. There is some limited evidence for lack of oxidative DNA damage from normal human GBF exposure. The studies of populations in regions where GBF spraying occurred do not provide clear evidence correlating exposure to chromosomal effects such as aberrations or induction of micronuclei. The single study result of DNA damage comet effects in a population presumably exposed to GBF aerial spraying might well have been due to abnormally high toxic exposures to the GBFs rather than a DNA-reactive mechanism and does not indicate genotoxic risk to humans under normal exposure conditions.

An earlier review of a very extensive number of experimental genotoxicity studies of glyphosate and GBFs concluded that there is a convincing weight of evidence supporting the lack of genotoxic potential for both glyphosate and typical GBFs in core gene mutation and chromosomal effect endpoints and that observations of DNA damage effects were likely to be secondary to toxicity (Kier and Kirkland 2013). This earlier review concludes that the lack of genotoxic hazard potential evidenced by core gene mutation and chromosomal effect studies, coupled with the very low human and environmental species systemic exposure potential, indicate that glyphosate and typical GBFs present negligible genotoxicity risk. A subsequent review of experimental rodent carcinogenicity studies did not indicate that glyphosate was associated with carcinogenicity (Greim et al. 2015) which supports the conclusion that glyphosate does not have DNA-reactive genotoxic properties. A review of human and environment genotoxicity biomonitoring studies does not indicate any significant evidence to contradict these conclusions.
